# Sweyr-James-MacLeod syndrome: a case report

**DOI:** 10.1016/j.ijscr.2025.112054

**Published:** 2025-10-10

**Authors:** Doaa Abo hamza, Sawssan Ali, Maha Alshahen, Zyad Al-Frejat

**Affiliations:** a Pediatric Hospital, Damascus University, Damascus, Syria; b Aleppo University, Aleppo, Syria; c Damascus, Syria; d Department of Radiology, Damascus University, Syria

**Keywords:** Swyer-James-MacLeod syndrome, Unilateral hyperlucency, Bronchiectasis, Lobectomy, Pediatric, Case report

## Abstract

**Introduction:**

Swyer-James-MacLeod syndrome (SJMS) is a rare, acquired pulmonary disorder predominantly affecting children, characterized by unilateral hyperlucency of the lung, bronchiectasis, and recurrent respiratory symptoms. Timely diagnosis and intervention are critical to prevent long-term morbidity.

**Case presentation:**

We present the case of an 11-year-old boy with a four-month history of recurrent productive cough, yellow sputum, and progressive respiratory distress. Despite multiple courses of antibiotics and expectorants, there was no clinical improvement. Chest X-ray and computed tomography revealed left lung hyperlucency, segmental bronchiectasis in the left lower lobe, and compensatory hyperinflation of the right upper lobe. Following the failure of conservative management, a left lower lobectomy was performed. Histopathology confirmed severe bronchiectasis with fibrosis and chronic inflammation, consistent with SJMS. The patient's symptoms improved significantly postoperatively.

**Clinical discussion:**

This case highlights the importance of considering SJMS in children presenting with chronic respiratory symptoms and unilateral hyperlucency on imaging. Surgical intervention may be warranted in patients with localized disease unresponsive to medical therapy.

**Conclusion:**

Early recognition and management of SJMS are essential to prevent irreversible lung damage. Lobectomy can be an effective treatment in selected cases with localized, severe disease.

## Highlights

• Swyer-James-MacLeod syndrome (SJMS) is a rare cause of unilateral hyperlucent lung in children.
• Diagnosis is often delayed due to nonspecific symptoms and overlap with more common pulmonary diseases.
• High-resolution computed tomography is essential for accurate diagnosis and disease localization.
• Surgical intervention, such as lobectomy, should be considered for severe, localized, and refractory cases.
• Early recognition and management of SJMS may prevent long-term respiratory complications.

## 1 Introduction

Swyer–James syndrome (SJS), also known as Swyer–James–MacLeod syndrome, is a rare, acquired pulmonary disorder characterized by unilateral hyperlucency of the lung, resulting from postinfectious bronchiolitis obliterans during early childhood. The disease is thought to arise following severe lower respiratory tract infections—commonly due to adenovirus—which lead to obliteration of the small airways, reduced vascularity, and impaired alveolar development. The condition was first described simultaneously in the 1950s by a respiratory physician William Mathieson Macleod in England (1954) [1], and by a physician Paul Robert Swyer and a radiologist George James in Canada. Clinically, patients may present with chronic productive cough, dyspnea, and recurrent pulmonary infections [2]. However, the syndrome often remains undiagnosed until imaging studies are performed, revealing the hallmark radiographic features of a hyperlucent lung with air trapping and possible bronchiectasis [3]. Differential diagnoses include congenital lobar emphysema, unilateral emphysema, and pulmonary hypoplasia, making accurate diagnosis challenging in pediatric patients presenting with non-specific respiratory symptoms. This case is significant due to the rarity of Swyer–James syndrome presenting with progressive and refractory respiratory symptoms requiring surgical intervention in a pediatric patient. The diagnosis is often delayed because of non-specific clinical features and overlapping radiological findings with other pulmonary conditions. Our case highlights the importance of considering Swyer–James syndrome in the differential diagnosis of unilateral hyperlucency and recurrent infections in children. It also underscores the role of lobectomy as a definitive treatment option in selected cases with localized disease and failure of conservative management. While most cases are managed conservatively, surgical resection may be indicated in patients with localized bronchiectasis and persistent symptoms unresponsive to medical therapy. In this report, we present a case of an 11-year-old male with Swyer–James syndrome involving the left lung, who underwent successful surgical management after failing to respond to prolonged medical treatment. This case emphasizes the importance of early recognition and appropriate surgical intervention in selected patients to prevent further lung damage and improve clinical outcomes.

## 2 Methods

The case has been reported in line with the Surgical Case Report (SCARE) 2025 criteria [4].

### 2.1 Case presentation

An 11-year-old boy has been admitted to the hospital with recurring productive cough with yellow sputum and respiratory distress for the past four months. Treatment with antibiotics and expectorants did not yield improvement. The child has no significant medical history, nor is there a positive family history of pulmonary diseases.

### 2.2 Clinical examination

On examination, the child exhibited mild tachypnea with a respiratory rate of 30 breaths per minute, a heart rate of 80 beats per minute, blood pressure of 100/60 mmHg, and oxygen saturation of 95 % on room air. Auscultation revealed a significant decrease in breath sounds at the left lung base, accompanied by widespread wheezing bilaterally. Other clinical findings were within normal limits.

### 2.3 Laboratory investigations

Laboratory results indicated a white blood cell count of 11,300/mm^3^, hematocrit of 35 %, and a platelet count of 498,000/mm^3^. Electrolytes, liver enzymes, and kidney function tests were all within normal ranges.

Preoperative pulmonary function tests demonstrated significant impairment: FVC 1.10 L (58 % predicted), FEV1 0.76 L (44 % predicted), FEV1/FVC 67.3 % (74 % predicted), PEF 1.82 L/s (47 % predicted), and FEF25–75 0.53 L/s (26 % predicted).

### 2.4 Radiological findings

**Fig. 1:**
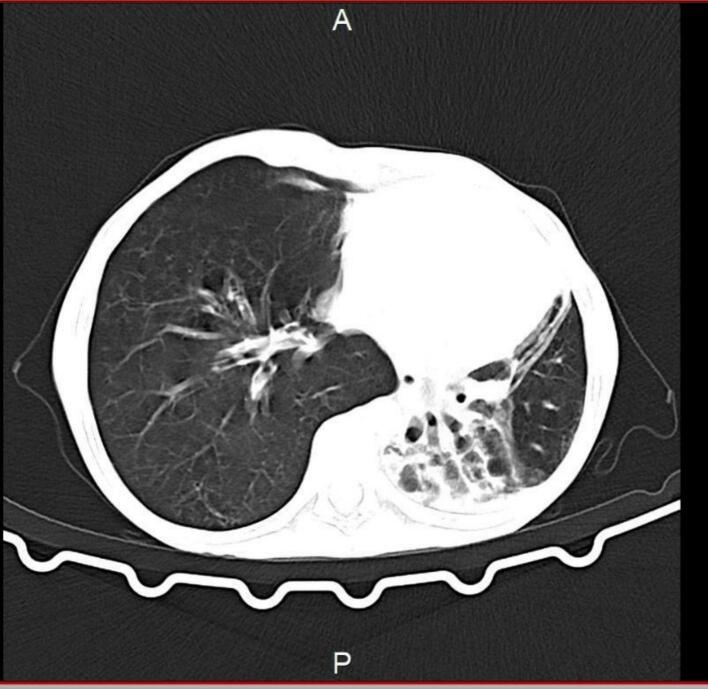
Axial chest CT shows severe volume loss and bronchiectasis of the left lung, with compensatory hyperinflation of the right lung—findings consistent with Swyer-James-MacLeod syndrome.

Imaging evaluation was performed with high-resolution computed tomography (HRCT), which provided definitive diagnostic information. HRCT revealed anatomically normal mediastinal vessels, with focal and segmental bronchiectasis in the left lung and consolidative changes in the left lower lobe. The study demonstrated unilateral hyperlucency of the left lung, associated bronchiectasis, and compensatory hyperinflation of the contralateral lung (Fig. 1). A diagnostic-quality preoperative PA chest radiograph was not available from the archive, and we acknowledge this as a limitation.

### 2.5 Surgical intervention

The patient underwent an open left thoracotomy for left lower lobectomy. Intraoperatively, the left lower lobe was markedly atrophic and destroyed, without major pleural adhesions or vascular anomalies. The lung tissue fragment measured 10 × 7 × 5 cm and showed markedly bronchioles surrounded by fibrosis and prominent inflammatory reaction (bronchiectasis).

### 2.6 Postoperative management

Postoperatively, the child was managed with IV ceftriaxone for 5 days, maintained NPO with IV fluids initially, and had a chest tube inserted which was removed on day 4 after cessation of air leak. Oral feeding was reintroduced gradually once stable. A postoperative chest X-ray confirmed re-expansion of the right lung. He was discharged on day 7 in good condition and remained asymptomatic at 6-month follow-up.

## 3 Discussion

Swyer-Jams-MacLeod syndrome (SJMS) is a rare pediatric pathological entity that affects the respiratory tract, usually post-infectious bronchiolitis obliterans during the infantile period or childhood [2]. Hyperlucency of the affected lung is the hallmark of SJMS resulting from hypoplasia or obliteration of pulmonary arteries and air trapping secondary to bronchiolar obstruction [5,6]. The diagnostic challenge seen in this SJMS is clearly highlighted in our case, as it can mimic more common chronic respiratory diseases, thus delaying accurate diagnosis and appropriate treatment [7].

Persistent cough, recurrent respiratory symptoms and insufficient response to conventional antibiotic therapy, are the main clinical presentation in this syndrome as seen in our patient. However, our patient demonstrated a notable localized bronchiectasis and extensive structural damage, which necessitated surgical intervention via lobectomy [8].

High resolution CT is the corner stone diagnostic method for diagnosing SJMS demonstrating unilateral hyperlucency, bronchiectasis, air trapping and compensatory hyperinflation of the collateral lung, characteristic finding aligning well with the existing literature. These findings underscore the importance of high resolution CT as a definitive imaging modality to identify subtle anatomical changes and guide subsequent management strategies [3].

Histopathological examination in SJMS reveals severe bronchiectasis with extensive fibrosis and chronic inflammatory infiltrates which mirrors the effects of longstanding airway obstruction and inflammation [9]. This becomes of high importance in complex and ambiguous cases which require surgical biopsy or resection, as histopatholgical confirmation can offer definitive evidence of SJMS [8].

When discussing management of SJMS, the main focus is symptomatic relief alongside infection control. Physiotherapy is crucial for preventing disease progression. Surgical intervention, as indicated in our patient, is reserved for severe, localized bronchiectasis and irreversible pulmonary destruction unresponsive to conservative treatment. This surgical approach significantly improved our patient's respiratory symptoms and quality of life, emphasizing the therapeutic potential of lobectomy in carefully selected cases. [7].

A limitation of this report is the absence of a diagnostic-quality preoperative chest radiograph. However, high-resolution CT provided clear evidence of bronchiectasis, unilateral hyperlucency, air-trapping, and vascular pruning, which are well-established diagnostic features of Swyer–James–MacLeod syndrome and sufficient for surgical planning.

## 4 Conclusion

Clinicians should maintain a high index of suspicion for SJMS in pediatric patients presenting with chronic, recurrent respiratory complaints and unilateral lung hyperlucency on imaging. Early recognition through comprehensive clinical evaluation, detailed radiological studies, and timely surgical intervention in refractory cases can significantly alter disease outcomes, reducing long-term morbidity associated with SJMS [1,2].

## CRediT authorship contribution statement

All authors contributed in collecting the data and writing and revising the manuscript.

## Informed consent

Written informed consent was obtained from the patient's parents for publication of this case report and any accompanying images. A copy of the written consent is available for review by the Editor-in-Chief of this journal.

## Ethical approval

The ethical approval for this study was waived by Damascus University ethics committee.

## Guarantor

Dr. Zyad Al-Frejat.

## Funding

None.

## Declaration of competing interest

The authors declare that there is no conflict of interest.
